# ROBOTIC SURGERY: BIOETHICAL ASPECTS

**DOI:** 10.1590/0102-6720201600040018

**Published:** 2016

**Authors:** Rodrigo SIQUEIRA-BATISTA, Camila Ribeiro SOUZA, Polyana Mendes MAIA, Sávio Lana SIQUEIRA

**Affiliations:** 1 Postgraduate Program in Bioethics, Applied Ethics and Public Health, Federal University of Rio de Janeiro, Rio de Janeiro, RJ; Brazil; 2 Department of Medicine and Nursing, Federal University of Viçosa, Viçosa, MG; Brazil; 3 Nucleus of Medical Sciences Studies, Dynamics Faculty of Piranga Valley, Ponte Nova, MG, Brazil.

**Keywords:** Bioethics, Surgery, Ethics, Robotics

## Abstract

**Introduction:**

The use of robots in surgery has been increasingly common today, allowing the emergence of numerous bioethical issues in this area.

**Objective:**

To present review of the ethical aspects of robot use in surgery.

**Method:**

Search in Pubmed, SciELO and Lilacs crossing the headings "bioethics", "surgery", "ethics", "laparoscopy" and "robotic".

**Results:**

Of the citations obtained, were selected 17 articles, which were used for the preparation of the article. It contains brief presentation on robotics, its inclusion in health and bioethical aspects, and the use of robots in surgery.

**Conclusion:**

Robotic surgery is a reality today in many hospitals, which makes essential bioethical reflection on the relationship between health professionals, automata and patients.

## RESUMO

**Introdução:**

A utilização de robôs em procedimentos cirúrgicos tem sido cada vez mais frequente na atualidade, o que permite a emergência de inúmeras questões bioéticas nesse âmbito.

**Objetivo:**

Apresentar revisão sobre os aspectos éticos dos usos de robôs em cirurgia.

**Método:**

Realizou-se revisão nas bases de dados Pubmed, SciELO e Lilacs cruzando-se os descritores "bioética", "cirurgia", "ética", "laparoscopia" e "robótica".

**Resultados:**

Do total de citações obtidas, selecionou-se 17 artigos, os quais foram utilizados para a elaboração do artigo. Ele contém breve apresentação sobre a robótica, sua inserção na saúde e os aspectos bioéticos da utilização dos robôs em procedimentos cirúrgicos.

**Conclusão:**

A cirurgia robótica é uma realidade, hoje, em muitas unidades hospitalares, o que torna essencial a reflexão bioética sobre as relações entre profissionais da saúde, autômatos e pacientes.

## INTRODUCTION

*"First Law: A robot may not injure a human being or, through omission, allow a human being to come to harm; Second Law: A robot must obey the orders given by human beings except where such orders would conflict with the First Law; Third Law: A robot must protect its own existence as long as such protection does not conflict with the First and/or Second Law".*

Asimov I. Eu, robô^4^

In health, the development of robots was to assist some tasks considered basic. The hospital support robotic system Helpmate Pyxis Corp., San Diego, California, USA, for example, moves transporting drugs, food, utensils and other equipment. It can be effective for all hospital areas, and allows professionals to perform other functions that have irreplaceable role. Already robots RX and AHC, both of McKesson company of San Francisco, California, USA, are designed to prepare drugs for enteral and parenteral application^12^. However, the surgical field has been taking advantage of these new technologies^1^, highlighting the use of modern robotic systems composed of visual device - for which there is control of camera movement - and motor device, responsible for surgical instruments. In some cases, there is a supplementary verbal command system which enables the control equipment by surgeon^24^.

At present, some models are important and can become essential in the coming medical practice, such as the AESOP Robotic Surgical System (Automated Endoscopic System for Optimal Positioning), built by the company Computer Motion Inc., of Santa Barbara, California, USA^12^. There are also robotic models such as the da Vinci, the Intuitive Surgical Inc., Sunnyvale, California, USA, and the ZEUS Robotic Surgical System, built by the company Computer Motion Inc., Goleta, California, USA with widespread use in laparoscopic, thoracoscopic and cervicoscopic operations^20^ ^,^ ^21^ ^,^ ^35^. Indeed, the robots - today - have contributed to greater precision in surgical procedures, collaborating, for example, to expand the three-dimensional field of view in interventions that require greater surgeon skill, as noted in colorectal videosurgeries and the arterial anastomosis in heart^3^ ^,^ ^20^.

Exceeding these technological boundaries has allowed significant innovation in surgical activity, generating the need for new ways of evaluating its implementation, especially in the technical, legal and bioethic fields^25^ ^,^ ^29^. Ethically, many questions have been raised - involving the relationship between humans and machines - which require more detailed approach. It is possible, in this aspect, that one of the first successful recommendation for the consideration of ethical aspects of the use of robots is contained in the "Three Laws of Robotics"^4^ presented in the title of this essay. Although they have appeared in works of science fiction of Isaac Asimov - inscribed in technophilic and vainglorious vision of science, according to which the robots would be great allies of humans - they may be useful in the very near future, to the extent that the automata are increasingly present in everyday life, performing actions and assisting humans at work and home^7^ with the intention, in the near future, they become genuine "companions" of human beings. Take the example of BINA48, a robot that is able to learn, talk to people and express feelings^13^.

Another interesting composition involving robotics and ethics is the roboethics - a term coined by Gianmarco Veruggio^33,34^ - which is concerned primarily with human behavior in interactions with robots and other machines that hold artificial intelligence^2^ ^,^ ^15^. It is interesting to note that in recent movies She (2014, directed by Spike Jonze) and Eve (2012; directed by Kike Maíllo) contain good examples of ethical conflicts that could result from those relationships.

The interrelations between bioethics and robotics make up a very new field of study in the scientific literature; there are few publications directed to the topic that must be more explored and discussed^11^ ^,^ ^15^.

Thus, based on these preliminary considerations, this article presents a brief presentation on robotics - emphasizing their inclusion in health - as an initial step to address the ethical issues surrounding the use of robots in surgery.

## METHOD

Through DeCS site (www.decs.bvs.br), the descriptors "ethics", "bioethics", "robotics", "surgery" and "laparoscopy" were selected and combined to determine search strategies of articles published in journals (Table 1). The following descriptor arrangements were used: A) ethics + robotics; B) bioethics + robotics; C) ethics + robotic + surgery; D) bioethics + robotics + surgery; E) ethics + robotic + laparoscopy; F) bioethics + robotics + laparoscopy, being applied in the databases Lilacs, PubMed, Scielo and Scielo BR with search deadline until June 30, 2015. The selected descriptors were searched in Portuguese in all databases except PubMed, in which was used English (Table 1).

**TABLE 1:** Articles search strategies in databases

	Database			
Strategy	Lilacs	PubMed	Scielo	Scielo BR
A	1	101	1	1
B	1	8	0	0
C	0	26	0	0
D	0	2	0	0
E	0	9	0	0
F	0	1	0	0

Of total obtained citations, 17 articles were selected - using as criteria the existence in the text of ethical considerations directed to the use of robots in surgical procedures - which were used for the preparation of this text.

## RESULTS

### Robotics: concept and current state

The term robotics derives from "robota" Czech word meaning "servant" or "worker"^14^ ^,^ ^15^ ^,^ ^18^. It is known that the word was coined by Karel Capek^14^ in theatrical spectacle R.U.R. (Rossum's Universal Robots). The word was popularized only years later, through the works of Russian Isaac Asimov, responsible for making the "Three Laws of Robotics"^4^ which, in fiction, standardize the robot´s behavior^14^ ^,^ ^18^. The laws allowed to these machines some free will, inspiring writers and directors (re)elaborate them in a variety of ways, as can be seen in films such as Star Wars (1977, directed by George Lucas) and AI - Artificial Intelligence (2001, directed by Steven Spielberg).

From fiction to real life, the application of robots started in the industry when General Motors introduced the Unimate^14^, device to prevent damage to the workers, replaced them in some functions in the car assembly line.

Not restricted to industry, from the 60s, robotics was present in different scenarios, from employment to explore the ocean depths to the use of rescue missions^14^ ^,^ ^18^. The applicability of these machines is vast, and this is evidenced by the formats in which are configured: it can be seen them as automated arms, mobile or telerobotics, and may be active, semi-active or passively handled^14^. The actively controlled devices perform their actions according to predetermined schedules; semi-actively or passively controlled devices reflect the physical movement of the controller, minimizing or maximizing the strength and range, allowing to perform actions that would be impossible without this aid. Moreover, are already available robots with locomotive capacity to develop reactive behaviors, for example obstacles, and build an evolutionary learning to adapt to different environments, whose refinement could produce important advances in robotics^26^.

### The use of robots in healthcare

Robots are already used in health care in surgical procedures for years. One of the first devices used in surgery was the forerunner of Neuromate - approved in 1999 by the Food and Drug Administration (FDA) - created to perform stereotactic brain biopsy accurately at 0.05 mm^14^. Then came the Robodoc - saw used in hip prosthesis replacement operation - ACRobot - used in knee operations - and RX-130 - used in operations in temporal region^14^. Currently, robots assist minimally invasive operations for interatrial communication correction^21^.

Evolving a little more in the field of surgery, was the telerobotics insertion by developing the da Vinci Surgical System - in which the surgeon performing the procedure through a console (or two consoles when performed by two surgeons), which controls three or four mechanical arms remotely (Systems da Vinci S, Si and the most current, Xi model) - and the surgical system Zeus - which made possible the realization of laparoscopic cholecystectomy in a patient who was in Strasbourg, France, by surgeon located in new York, USA. By allowing conducting operations without the physical presence of the surgeon's hand to the patient, the Advanced Research Projects Agency of the Pentagon's Defense envisions using this technology in battle fields^14^, minimizing medical risk if exposed to dangerous front. Both the Zeus system and the da Vinci obtained FDA approval.

With robotics progress in health, and observing the current literature, we note that the implementation of robots has been beneficial in surgical operations of the head and neck, gastrointestinal, gynecological, cardiac and urologic procedures^18^ ^,^ ^23^.

Increasing the degree of complexity of operations and the consequent difficulty of training some techniques by learners - particularly the ethical and legal issues involved - contributed to the invention of simulators that generate, with the aid of computer and robotic techniques, virtual reality environments^29^. Basic tasks are simulated - as sutures, hemostasis and dissection - but also large procedures such as cholecystectomy and gastrofundoplications, among others. Simulators promise intimate approach to reality, contributing to the improvement of surgical techniques and in parallel to minimize the risks to real patients. Currently, the training system and accreditation of surgeons, most used, is the Mimic System Mimic Technologies of Seattle, Washington, USA

Regarding non-surgical treatments, note the use of robotic devices primarily in physical medicine and reabilitation^6^ ^,^ ^16^ ^,^ ^17^ ^,^ ^30^. In patients who have experienced stroke, the use of robots has helped to relearn the movement of certain muscular groups^6^ ^,^ ^16^ ^,^ ^17^. Swinnen et al^30^ in their review article, noted that further investigation is still needed to conclude something about the applicability of robotics in physical and rehabilitation medicine^6^ ^,^ ^16^ ^,^ ^30^.

### Bioethical issues in the use of robots

The progress of robotics, along with the favorable results obtained with the use of robots in operations, has raised ethical discussions on the possible limits on the use of automata in operative environment^16^.

The question of responsibility - the indication and execution of surgery - does not differ in substance from medical situations in which there is no involvement of robots. In fact, the professional is responsible for robotics participation in surgical procedures, which can be defended using disparate contemporary bioethical currents^22^; may be mentioned, by way of example, the obligation to not harm (the principle of non-maleficence/principialist current); to act for the benefit of the patient (principle of beneficence/principialist current); to consider self-determination (principle of respect for autonomy/principialist current); to calculate the consequences (consequentialism/utilitarian current); and to maintain moral patient care (ethics of care)^22^. Similarly, in ethical terms, the current Medical Code of Ethics states that^10^:

The physician is prohibited:

Art. 3. Failing to take responsibility for medical procedure indicated or attended, even when several doctors have assisted the patient.

[...]

Art. 6. Assign their failures to third parties and the occasional circumstances, except in cases where it can be duly substantiated.

(CFM, 2016) 10.

The situation becomes more complex when is added to bioethics interface/robotics issues on the telemedicine^31^. Telemedicine can be understood as the "provision of health services for remote telecommunication", which "includes interactive consultation and diagnostic services"^8^. Through this service, health care professionals may use technological devices for communication and the exchange of important information to promote the health of individuals and populations, even when the objective involves research and heath evaluation^31^.

The Federal Medical Council regulates the provision of services through telemedicine since 2002, in Resolution No. 16439, which provides for the goals of this healthcare modality, appropriate methodologies, health research and appropriate technological infrastructure, including ethical aspects, with emphasis on confidentiality, professional secrecy and privacy, among others^9^:

Art. 2 - The services provided by the Telemedicine should have the appropriate technological infrastructure, relevant and comply with the technical standards of CFM relevant custody, handling, data transmission, confidentiality, privacy, and assurance of professional secrecy.

(CFM, 2002)^9^

The resolution brings manifestation in order to comment on the responsibility in Medical Code of Ethics^10^, as highlighted below:

Art. 4 - The professional responsibility of care is up to the doctor of the patient. The other involved jointly liable to the extent that contribute to any damage to him.

(CFM, 2002)^9^

Such resolutions must be considered in the case of operations using robots, especially when the use of artifacts is held at a distance, in which case the procedure becomes part precisely the scope of telemedicine.

Other bioethical issues - but that would flee the scope of this article - could be briefly mentioned: 1) emergency of machines with possibilities genuinely intelligent^19,27^ - with the advancement of techniques and artificial intelligence, especially towards strong AI^19,27^ - which would raise the question of what level of ethical consideration these thinking beings deserve; and 2) the use of robots in reprehensible acts - the legal and ethical point of view - for example, the action in order to take human lives and not human (wars, terrorism, etc.) ^5^ ^,^ ^11^ ^,^ ^28^ . In this sense, the adoption - indeed - of these "Three Laws of Robotics" ^4^ ^,^ ^5^ ^,^ ^28^, could become an interesting - and perhaps vital - prerequisite for the use of these artifacts^11^ ^,^ ^15^.

From these questions, it is necessary the effort to unite the two cultures - bioethics and robotics - so problems could be understood in their entirety and complexity^34^.

## CONCLUSION

Robots - coming art/fiction - have become increasingly present in contemporary reality. In fact, you can see them working in places where humans cannot go - because of its biological limitations - and helping women and men in different fields of knowledge, as the area of ​​health.

In this regard, there is emphasis on the advances in the use of these devices in surgical procedures, with good results in different types of interventions. In this scenario, the bioethical debate on robotic surgery - still incipient in educational environments and health research - becomes very salutary, to provide support to decision-making in situations where the robots are partakers of care actions to humans.

## References

[1] Abdalla RZ (2012). Cirurgia robótica, devo abrir mão. Arq Bras Cir Dig.

[2] Anderson M, Ishiguro H, Fukushi T (2010). "Involving interface": an extended mind theoretical approach to roboethics. Account Res.

[3] Araujo SEA (2008). Video cirurgia colorretal com assistência robótica o próximo passo?. Rev Bras Coloproct.

[4] Asimov I (2009). Eu, robô.

[5] (2011). Autores não listados Beyond the bomb. Nature.

[6] Belda-Lois JM (2011). Rehabilitation of gait after stroke: a review towards a top-down approach. J NeuroEngineering and Rehabilitation.

[7] Bento LA, Calvo PRS (2013). Quando a vida imita a arte a bioética dos homens-máquinas. Revista Bioethikos.

[8] Biblioteca Virtual de Saúde (2016). Descritor: Telemedicina.

[9] Conselho Federal de Medicina (2002). Define e disciplina a prestação de serviços através da Telemedicina. RESOLUÇÃO CFM nº 1.643.

[10] Conselho Federal de Medicina (2009). Código de Ética Médica. RESOLUÇÃO CFM nº 1.931.

[11] Fabris A, Bartolommei S, Datteri E (2008). Ética y robótica Eikasia. Rev Filosofía.

[12] Felippe de Souza JAM (2005). Robótica - Cap. 5: Robôs na Medicina.

[13] (2016). Hanson Robotics. Interview with a robot: BINA48.

[14] Hockstein NG, Gourin CG, Faust RA, Terris DJ (2007). A history of robots from science fiction to surgical robotics. J Robotic Surg.

[15] Ishihara K, Fukushi T (2010). Introduction roboethics as an emerging field of ethics of technology. Account Res.

[16] Kalra L (2010). Stroke Rehabilitation 2009: Old Chestnuts and New Insights. Stroke.

[17] Knecht S, Hesse S, Oster P (2011). Rehabilitation after stroke. Dtsch Arztebl Int.

[18] Olavarrieta JRL, Coronel P, Pérez YO (2007). Historia, evolución, estado actual y futuro de la cirurgía robótica. Rev Facultad Med.

[19] Pereira IS (2006). Eu, robô e a inteligência artificial forte o homem entre mente e máquina. Ciênc Cogn.

[20] Poffo R (2013). Cirurgia robótica em Cardiologia um procedimento seguro e efetivo. Einstein (São Paulo).

[21] Poffo R (2012). Cirurgia minimamente invasiva robô assistida na correção da comunicação interatrial. Rev Bras Cir Cardiovasc.

[22] Rego S, Palácios M, Siqueira-Batista R (2014). Bioética para profissionais da saúde. 1ª reimp.

[23] Rodriguez E, Chitwood WR (2009). Robotics in cardiac surgery. Scand J Surg.

[24] Sant'anna RT (2004). Emprego de sistemas robóticos na cirurgia cardiovascular. Rev Bras Cir Cardiovasc.

[25] Santos EG (2011). Super especialização na cirurgia geral problema ou solução?. Rev Col Bras Cir.

[26] Selvatici AHP, Costa AHR (2007). Aprendizado da coordenação de comportamentos primitivos para robôs móveis. Sba Controle & Automação.

[27] Shimoda M (2013). Brain, mind, body and society: autonomous system in robotics. J Int Bioethique.

[28] Singer PW (2011). Military robotics and ethics a world of killer apps. Nature.

[29] Skinovsky J, Chibata M, Siqueira DED (2008). Realidade virtual e robótica em cirurgia aonde chegamos e para onde vamos?. Rev Col Bras Cir.

[30] Swinnen E (2010). Effectiveness of robot-assisted gait training in persons with spinal cord injury a systematic review. J Rehabil Med.

[31] Mariani AW, Pêgo-Fernandes PM (2012). Telemedicine a technological revolution. São Paulo Med J.

[32] Valdiero AC (2005). Controle de Robôs Hidráulicos com Compensação de Atrito.

[33] Veruggio G, Operto F (2008). Roboethics: Social and ethical implications of robotics, in Springer handbook of robotics, edited by B. Siciliano and O. Khatib, pp. 1499 1524,.

[34] Mushiaki S (2013). Ethica ex machina: issues in roboethics. J Int Bioethique.

[35] Zorron R, M D, Madureira F, Jamel N (2005). Videocirurgia robótica estudo clínico prospectivo na colecistectomia laparoscópica. Rev Col Bras Cir.

